# Progress in Medicine

**Published:** 1899-04-22

**Authors:** 


### Progress in Medicine.

### DISEASES OF THE THYROID GLAND.

(*Continued from page 48.*)

**hyroid Treatment of Sporadic Cretinism.**—Koplik[4] discusses the ultimate results of treatment of cases of sporadic cretinism with thyroid gland. The immediate results are always striking, the swelling of the face diminishes, the anæmia improves, the tongue is not so large, and the infant notices its mother. The ultimate results depend in great measure on whether the cases are first treated in infancy, childhood, adolescence, or adult life. The best results occur where the child is only a few months old. Such cases may be recognised by the stupidity of the infant, the low internal temperature, the shortness of the limbs, the enormous tongue, and the coarse cry. In these cases an almost normal intellectual and bodily development may be secured by continuous administration of thyroid. After this age it is very rare to find a complete cure—the initial effect is marvellous, but the older the patient the worse are the ultimate results, the more lacking are some of the mental and physical essentials which go to make up the normal individual. The mental condition in particular always point towards a childishness, a very limited vocabulary, uncertainty of gait and weakness in extremities. Similarly, the older the child, the slower is the rate of growth obtained by thyroid treatment. If treatment were suspended there was a speedy relapse. In discussing the paper Jacobi said that one-third of the cretins he had seen were brachycephalous, the result of premature synostosis of the basicranial axis. This condition could not be changed by thyroid medication, and was probably the cause of the fact that the older the patient the less striking the ultimate results of treatment.

**Pharmacological Action of the Thyroil Gland.**—Hutchison[5] discusses the present state of our knowledge of the physiological effects of the administration of thyroid and the bearing of that knowledge on its use as a medicinal agent. The administration of thyroid increases oxidation in the body. The products of the disintegration of the nitrogenous tissues appear in the urine almost entirely in the form of urea, uric acid, and xanthin bases, being neither regularly or appreciably increased; whilst the products of fat destruction are eliminated as carbonic acid by the lungs and water by the kidneys. The amount of water excreted by the kidneys is largely increased. An important practical question, particularly in regard to the question of the treatment of obesity by thyroid, is whether thyroid acts most powerfully on proteid or on fatty tissues. Hutchison is of opinion that probably the increased elimination of urea which is observed at the commencement of thyroid feeding is due to destruction of circulating proteid, and that the fixed proteid tissues are only attacked when the store of fat is appreciably diminished. The practical deduction is that in treating obesity by thyroid feeding the diet should not be too much restricted, and that nitrogenous matter should be abundantly represented in it. It is not yet determined how thyroid acts, whether on the tissues directly or through the intervention of the nervous system; in favour of the latter view is the very minute quantity of the substance which is sufficient to produce powerful effects. Whatever the exact way in which the influence takes place, its essential nature would seem to consist in a hastening on of the life history of the cells. The reverse of this process occurs typically in myxœdema, where many of the cells never seem to reach a mature stage; thus the subcutaneous tissue becomes filled with cells in an embryonic stage, and when the old hairs fall out the cell division which should lead to the formation of new ones ceases to occur. This action of hastening on the life history of cells explains its action in backwardness of growth in children and the good effect it sometimes exerts in such diseases as psoriasis and ichthyosis, where the final death and casting off of the cells seems much delayed. Simple goitre can often be cured by the administration of thyroid; a fact which may be explained by supposing that the hypertrophy of the gland is due to a great demand being put on it. Bruns has confirmed this view by observation on dogs with goitre. He examined a piece of the gland microscopically, and found that it presented the characters of a gland which was over-secreting. After thyroid treatment the goitre had almost disappeared, and on examining a fresh portion he found that the gland had now resumed its normal characters. The occurrence of glycosuria as a result of thyroid administration has frequently been observed, and recent experiments tend to show that this glycosuria may be of the alimentary type. Thus Bettmann found that if after a week of thyroid feeding one gives a patient 100 grains of sugar, glycosuria results in about half the cases. Increased rapidity of the heart's action is perhaps the most constant of the effects observed from thyroid administration; in fact, the action of the drug can hardly be said to be manifested unless the pulse rise ten or more beats a minute. And with this increased rapidity there is often irregularity, palpitation, or even threatenings of failure. This specific effect of the thyroid on the heart renders caution necessary in administration of the drug to patients with cardiac debility, especially in cases of obesity, where the fatty change may also have affected the heart. It also suggests its use in cases of functional brachycardia. It has been found that the active part of the thyroid has no effect on the blood pressure; the slight fall in pressure in patients who are under the influence of the thyroid seems to be due to enfeeblement of the heart rather than to any peripheral dilatation of the blood vessels. Small doses of thyroid produce no effect on the blood of healthy persons, whilst large ones cause an increased destruction of red blood corpuscles, and a certain amount of leucocytosis. In myxœdema, on the other hand, the stimulus which thyroid feeding gives to all growth and division is manifested in the blood by a rise in the number of corpuscles. The active constituent of the thyroid is excreted entirely by the kidney; this excretion appears to be gradual only, for the action of the drug continues often for several days after the administration of it has ceased. Whatever the exact form in which thyroid is given, whether the dried gland or the liquor thyroidei of the Pharmacopœia, it is best to give it in small doses and frequently. The extent of the reaction in any particular person cannot be foretold. The symptoms produced by over-action are headache and pains in the limbs, nausea, diarrhœa, palpitation, and rapid pulse. Of these, the alimentary group are probably to be explained by the presence of putrefactive substances, and are absent if the preparation is fresh; whilst the headache, pains in the limbs, and cardiac symptoms are due to a specific action of the thyroid.

Fresh thyroid contains only one iodine-containing compound, namely, the colloid matter. This consists of a proteid part (a nucleo-proteid), combined with an organic compound of iodine (crude iodothyrin), and the latter compound is split off on hydrolysis of the colloid. The specific effects of the thyroid can be produced either by the colloid matter, as a whole, or of the iodothyrin split off from it. Further, the colloid matter contains all the activity of the thyroid, the extractives being incapable of producing any of the effects of the gland.

Blum's[6] researches on the iodine-containing substance in the thyroid confirm those of Hutchison. He finds it is a substance which can be split up into iodothyrin, and an albumen. The iodothyrin can be increased by administration of iodine-containing substances. Thus, the administration of potassium iodide will cause the thyroid gland to store it up in the form of iodothyrin; whilst, unlike the thyroid, the other organs of the body can retain the iodine only for a short time when it is again taken up into the circulation.

**Surgical Treatment of Exophthalmic Goitre.**—Doyen[7] says there are but two operations which may be done—both of which have the same end in view—thyroidectomy, and section of the cervical sympathetic. There is no great difference in the dangers of the two operations. The champions of the latter operation say that the tachycardia diminishes and disappears, and that the goitre and exophthalmus do so too, although more slowly than the tachycardia. Doyen, on the contrary, maintains that thyroidectomy produces radical results, not merely ameliorations; it suppresses, for instance, the tachycardia in from eight to ten days. In support of these arguments he cites two cases, one operated on eight and the other four years ago, both of whom are now perfectly well. Booth[8] records the cases of eight patients suffering with exophthalmic goitre who were treated by excision of one lobe. Five were cured, one died. One had been improved, and in the last no improvement had taken place in six months. The order of improvement was the following: First the goitre diminishes, next the nervous symptoms disappear, then the pulse rate and vasomotor phenomena improve, and the exophthalmus last of all. These results agree generally with those which have been reported; in about 80 per cent. a cure or improvement takes place. Fatal resuls occasionally occur; 13 are known to have occurred from 1890 to 1898, *i.e.*, in about seven per cent. of cases. The death takes place suddenly, either at the time of operation or soon afterwards, and the rapid onset of symptoms is as yet unexplained. Stokes[9] also records a successful case of partial excision of the thyroid for exophthalmic goitre.

[4] Koplik, New York Med. Jour., July 16, 1898. [5] Hutchison, Brit. Med. Jour., July 16, 1898. [6] Blum, Münch Med. Woch, Nos. 8 and 9. [7] Doyen, Rev. de Chir., Nov. 11, 1897. [8] Booth, Med. Rec., Aug. 13, 1898. [9] Stokes, Brit. Med. Jour., Oct. 29, 1898.

### DISEASES OF THE NERVOUS SYSTEM.

**Cerebro-spinal Fluid.**—Thomson, Hill, and Halliburton[1] have investigated the composition of a fluid which continuously dropped from the nose of a patient, and analysis of which showed it to be cerebro-spinal fluid. Cases of escape of this fluid after fracture of the cribriform plate of the ethmoid have been previously recorded, but not one in which the flow was spontaneous. The fluid was clear and colourless, of s.g. 1,005, slightly alkaline in reaction, and contained no cells or other deposit. It contained a trace of proteid, which was found to be a globulin. It reduced Fehling's fluid, but did not ferment with yeast or yield osazone crystals, or yield any evidence of creatinine. From the residue after evaporation characteristic crystals of pyrocatechin were produced. The fluid was slightly poorer in solids in the evening as compared with the morning's flow. Hill had previously shown that the secretion of the cerebro-spinal fluid, when the cranio-vertebral cavity is opened, depends entirely on the difference between the pressure in the cerebral capilliaries and that of the atmosphere, and that the former varies directly and absolutely with vena cava pressure. Experiments on this patient entirely confirmed this view, for it was found that compression of the abdomen and forced expiratory effort with closed glottis, both of which raise the vena cava pressure, accelerated the flow of fluid from the nose.

**Cerebral Activity and Sleep.**—During the last few years various observers have attempted to discover a physical basis for the different phases of cerebral activity and sleep, more particularly in amœboid activity of the nerve cells which build up the nervous system. Thus Lépine thinks that sleep is caused by a temporary shrinking of the extremities of the nerve cells causing interruption of contact and consequent suspension of function. Lugano[2] has recently made an important research into the question. He denies the existence of amœboid movement in its strict sense, and limits the movement to the gemmulæ (or thorn-like processes) of the protoplasmic processes of nerve cells, which exhibit little movements of propulsion and retraction sufficient to make and break contact with the gemmulæ of other neighbouring cells. He maintains that when a nerve cell is in a state of physiological excitement, all its connections do not come into play; and conversely, when it is at rest, as in sleep or in poisoning by morphine or chloroform, there occurs a general expansion of the gemmulæ with exhaustion of their contractility at the beginning and renewed capacity to contract at the end of sleep. In functional activity the great majority of possible connections are interrupted in order to impede the access of other stimuli which would be able to deviate or suppress the one which is being elaborated in the cell, and they are restored whenever the active state of the cell ceases in order to render possible the reception of new stimuli from other provinces. Thus, according to Lugano, cerebral activity involves a cutting off of the great majority of interneuronic connections, and the strengthening of the current traversing the few which are essential for the anatomy of the functional association.

**Antero-lateral Ascending Tract.**—Rosolimo[3] records a case in which the lower end of the spinal cord was crushed by a growth spreading from the lumbar vertebræ. The ascending degenerations were traced by Marchi's method. The antero-lateral tract was found to end partly in the posterior corpora quadrigemina, part of it ran in the fillet, and there was a partial degeneration in the region of the fourth cranial nerve; some ran into the substantia nigra, and the remainder into the globus pallidus. No fibres were traced into the cerebellum.

On the other hand Alexander Bruce,[4] by a similar method, has traced the tract from a case of compression of the cord in the lower cervical region. The antero-lateral ascending or ventral cerebellar tract was found to pass up the side of the medulla, hook round the outgoing fifth nerve, and then curve along the outer side of the superior cerebellar peduncle in an upward and backward direction, downwards towards the anterior medullary velum of the cerebellum, to end in the lingual lobule of the vermis almost entirely on that of the same side. A few of the fibres entered the inferior corpus quadrigemina on the same and on the opposite side, but the number of fibres which did so was altogether insignificant compared with the number which entered the lingual lobe. This description, for man, exactly agrees with that described by Loewenthal, Mott, and Tooth in the monkey. In the same case, the direct cerebellar tract was traced up the inferior cerebellar peduncle, then inward towards the superior vermis, and terminated on both sides of the lobus centralis, lobus monticuli, and to a slight extent in the lobus lingualis. The superior cerebellar commissure would appear to be mainly composed of decussating fibres from the two cerebellar tracts, and is, therefore, not analogous with the corpus callosum of the cerebral hemispheres.

**Agoraphobia,** or dread of an open space, is graphically described by Neale.[5] Agoraphobia is probably a more common affection than is generally supposed. Professional men probably suffer most, though merchants and commercial travellers are sometimes its victims. It seldom attacks poor people. The attack occurs suddenly, a fear of impending death, though with no giddiness or faintness, rather a feeling of collapse, as though one were being shut up like a crush hat or Chinese lantern, with a tendency to fall unless some support is at hand. Next in order comes the dread of an open space, called up by the sensation of vastness, infinity, and solitude. Then the inability to sit in the body of a church or crowded meeting, though the patient, as a rule, feels safe when near the door. Agoraphobia does not endanger life except under special circumstances—*e.g.*, if an attack comes on when the patient is on a ladder. An agoraphobic can generally be recognised by always carrying a stick or umbrella, which he uses to increase his base line of support. The affection is more often associated with dyspepsia than any other malady. The avoidance of overstrain and all excesses, a careful dietary, and tonics will generally conduce to a disappearance of this pathological "spook," it being in reality but a phantom disorder.

[1] Thomson, Hill, and Halliburton, Lancet, Mar. 4, 1899. [2] Lugnot, Rivesta di Path. Nerv., Aug., 1898. [3] Rosilimo, Neurol. Centralblaat, Oct. 15, 1898. [4] Bruce, Brain, Autumn, 1898. [5] Neale, Lancet, Nov. 19, 1898.

